# Multiparameter diagnostic model based on ^18^F-FDG PET metabolic parameters and clinical variables can differentiate nonmetastatic gallbladder cancer and cholecystitis

**DOI:** 10.1186/s12885-023-10599-7

**Published:** 2023-02-06

**Authors:** Can Li, Xiaohui Luan, Xiao Bi, Shengxin Chen, Yue Pan, Jingfeng Zhang, Yun Han, Xiaodan Xu, Guanyun Wang, Baixuan Xu

**Affiliations:** 1 Department of Nuclear Medicine, The First Medical Center, Chinese PLA General Hospital, 28 Fuxing Road, Haidian District, Beijing, 100853 China; 2 Graduate School, Chinese PLA General Hospital, 28 Fuxing Road, Haidian District, Beijing, 100853 China; 3 Department of Gastroenterology and Hepatology, The First Medical Center, Chinese PLA General Hospital, 28 Fuxing Road, Haidian District, Beijing, 100853 China; 4 Nuclear Medicine Department, Beijing Friendship Hospital, Capital Medical University, 95 Yong’an Road, Xicheng District, Beijing, 100050 China

**Keywords:** PET, Nonmetastatic gallbladder cancer, Cholecystitis, Multiparameter, Metabolic parameters, differential diagnosis

## Abstract

**Objective:**

To evaluate the diagnostic value of a multiparameter model based on ^18^F-fluorodeoxyglucose positron emission tomography (^18^F-FDG PET) metabolic parameters and clinical variables in differentiating nonmetastatic gallbladder cancer (GBC) from cholecystitis.

**Patients and methods:**

In total, 122 patients (88 GBC nonmetastatic patients and 34 cholecystitis patients) with gallbladder space-occupying lesions who underwent ^18^F-FDG PET/CT were included. All patients received surgery and pathology, and baseline characteristics and clinical data were also collected. The metabolic parameters of ^18^F-FDG PET, including SUVmax (maximum standard uptake value), SUVmean (mean standard uptake value), SUVpeak (peak standard uptake value), MTV (metabolic tumour volume), TLG (total lesion glycolysis) and SUVR (tumour-to-normal liver standard uptake value ratio), were evaluated. The differential diagnostic efficacy of each independent parameter and multiparameter combination model was evaluated using the receiver operating characteristic (ROC) curve. The improvement in diagnostic efficacy using a combination of the above multiple parameters was evaluated by integrated discriminatory improvement (IDI), net reclassification improvement (NRI) and bootstrap test. Decision curve analysis (DCA) was used to evaluate clinical efficacy.

**Results:**

The ROC curve showed that SUVR had the highest diagnostic ability among the ^18^F-FDG PET metabolic parameters (area under the curve [AUC] = 0.698; sensitivity = 0.341; specificity = 0.971; positive predictive value [PPV] = 0.968; negative predictive value [NPV] = 0.363). The combined diagnostic model of cholecystolithiasis, fever, CEA > 5 ng/ml and SUVR showed an AUC of 0.899 (sensitivity = 0.909, specificity = 0.735, PPV = 0.899, NPV = 0.758). The diagnostic efficiency of the model was improved significantly compared with SUVR. The clinical efficacy of the model was confirmed by DCA.

**Conclusions:**

The multiparameter diagnostic model composed of ^18^F-FDG PET metabolic parameters (SUVR) and clinical variables, including patient signs (fever), medical history (cholecystolithiasis) and laboratory examination (CEA > 5 ng/ml), has good diagnostic efficacy in the differential diagnosis of nonmetastatic GBC and cholecystitis.

## Introduction

As a relatively rare malignant tumour, the 5-year survival rate of patients with invasive stage III or IV gallbladder cancer (GBC) is estimated to be less than 5% [1, 2]. Resection is the best treatment for patients with clinically localized gallbladder cancer, providing the only chance for cure [3]. The clinical manifestations of GBC are usually nonspecific symptoms, including abdominal pain, anorexia, weight loss, jaundice, pruritus, and scleral icterus [4]. Therefore, sporadic GBC is most often found in patients who receive evaluation of symptoms related to gallstones or surgery [5]. Similarly, there are no highly sensitive or specific tumour markers for GBC diagnosis in laboratory examination, even though carcinoembryonic antigen (CEA) and carbohydrate antigen 19 − 9 (CA19-9) may be elevated and are often used in the management of GBC patients [6, 7]. Cholecystitis is a common benign disease in the biliary system that is usually caused by gallstones (and less often, biliary sludge) obstructing bile egress from the gallbladder [8]. Patients can experience abdominal pain, tachycardia, fever and other symptoms, accompanied by leukocytosis during the acute attack, and some chronic cholecystitis patients may only have slight signs [9]. Since the symptoms and signs of GBC and some cholecystitis patients are similar, and there is no special laboratory examination to distinguish them, imaging examination is extremely important. However, in some patients with GBC and cholecystitis, traditional imaging examinations (such as ultrasound [US], computed tomography [CT] and magnetic resonance imaging [MRI]) are very difficult to differentiate [10, 11], which may lead to changes in treatment strategies, especially for patients with early resectable GBC. Especially when GBC is diagnosed by traditional imaging examinations, it is very important to evaluate the stage to determine the patient's treatment strategies. However, traditional imaging examination has limitations in GBC staging, and ^18^F-fluorodeoxyglucose (^18^F-FDG) positron emission tomography/CT (PET/CT) could play a role.

^118^F-FDG PET/CT has proven its value in the management of patients with GBC; in particular, it may be helpful to detect local lymph node metastasis and distant metastasis [12]. In the current guidelines, ^18^F-FDG PET/CT is recommended to identify lymph node metastasis, distant metastasis and disease recurrence [13]. When the anatomical imaging is equivocal, ^18^F-FDG PET/CT can also be considered for diagnosis [14]. However, the accumulation of FDG in inflammatory lesions will also increase [15], which may affect the accuracy of ^18^F-FDG PET/CT in diagnosing patients with gallbladder-occupying lesions, especially for patients without metastasis. Therefore, in the clinical work of nuclear medicine, the characteristics of solitary gallbladder lesions with high FDG uptake are often confused. The purpose of this study was to first determine whether ^18^F-FDG PET metabolic parameters can differentiate nonmetastatic GBC from cholecystitis; second, we sought to determine whether we could more effectively differentiate nonmetastatic GBC from cholecystitis based on FDG metabolic parameters and clinical variables.

## Materials and methods

### Patients

**Fig. 1:**
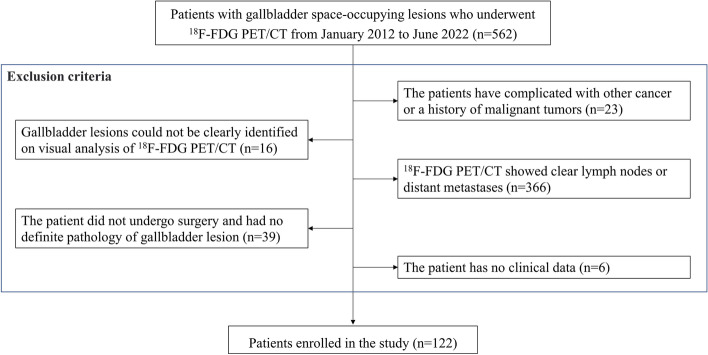
Flow chart

We collected patients with gallbladder space-occupying lesions who underwent ^18^F-FDG PET/CT from January 2012 to June 2022. The inclusion criteria were as follows: (1) no history of malignancy or complications with other cancers; (2) nonmetastatic GBC (no evidence of distant metastasis); and (3) GBC and cholecystitis who underwent surgery and were diagnosed by pathology. The exclusion criteria were as follows: (1) low-quality ^18^F-FDG PET/CT images and (2) no clinical data. The flow chart is shown in Fig. 1. The patient's clinical variables, including medical history, symptoms and signs, and laboratory examination (including WBC [white blood cell], LYM [lymphocyte], NEUT [neutrophil], CEA and CA19-9) were collected through medical records.

### Image acquisition

All patients were scanned with ^18^F-FDG PET/CT (Discovery 710, GE Healthcare, Germany/Biograph 64, Siemens Healthineers, Germany). Before intravenous injection of ^18^F-FDG (3.5–4.5 MBq/kg), the patient needed to fast for 6 hours, have blood glucose levels < 11.1 mmol/L, and rest in a quiet waiting room for at least 20 minutes. After 60 minutes of injection, images were collected from the skull base to the upper femur in free-breathing mode. The parameters of PET were 3-dimensional mode, 2-2.5 min/bed (30% overlap), 4–5 beds/person, three iterations, 21 subsets, and Gaussian filter half-height width = 4.0 mm. The low-dose CT (LDCT) parameters included voltage = 120–140 kV, current = 100 mAs, rotation = 0.8, layer thickness = 3–5 mm, and pitch = 1 [16]. The images were reconstructed with CT attenuation correction (AC) using the ordered subset expectation maximization algorithm (OSEM).

### Image analysis

Two experienced nuclear medical doctors (LC and WGY) both read all cases separately and reached a consensus on the commercial workstation (Advantage workstation 4.6, GE HealthCare) when the patient's clinical data were not clear. Gallbladder regions with abnormal ^18^F-FDG uptake on PET were defined as lesions. The two-dimensional region of interest (ROI) was manually delineated according to the boundary of tumour lesions on each horizontal axis CT image to form a three-dimensional volume of interest (VOI). There are some essential differences between the two PET/CT systems in machine design and scintillation detection, which may confuse the SUVmax measurement results, at least to some extent [17]. To solve this issue, we retrospectively calculated the SUVmean of the liver parenchyma in 126 patients for whom the original PET/CT images were available (GE Discovery 710, *n* = 100; Siemens Biograph 64, *n* = 26) [18]. To measure normal liver parenchyma activity, 3 nonoverlapping spherical 1-cm^3^-sized VOIs were drawn in the normal liver on axial PET images. There were no significant differences in terms of SUVmean-liver among the 2 PET/CT scanners (GE Discovery 710, 2.36 ± 0.32 vs. Siemens Biograph 64, 2.34 ± 0.34, *P* = 0.860).

The parameters of PET/CT included SUVmax (maximum standard uptake value), SUVmean (mean standard uptake value), SUVpeak (peak standard uptake value), MTV (metabolic tumour volume), TLG (total lesion glycolysis, SUVmean×MTV), and SUVR (tumour-to-normal liver standard uptake value ratio, SUVmax of the tumour/SUVmean of the normal liver parenchyma). MTV was measured from attenuation-corrected ^18^F-FDG-PET images by two nuclear medicine physicians (WGY and LC) with 5 and 10 years of experience, respectively, in making these measurements. The 40% threshold of SUVmax in the lesion was used to calculate the MTV [19].

### Statistical analysis

The statistical analysis was performed using commercially available software (IBM SPSS Statistics 24, IBM, Armonk, NY; and R software program, version 4.0.2, Bell Laboratories, USA). Quantitative data are described as the means ± SD (standard deviation) for continuous variables, and qualitative data are described as the numbers of cases and percentages [n (%)] for categorical variables. Student’s *t* test, the Mann‒Whitney test and the chi-squared test were used to compare ^18^F-FDG PET metabolic parameters, baseline characteristics and clinical variables between nonmetastatic GBC and cholecystitis. The area under the receiver operating characteristic (ROC) curve was calculated to assess the predictive value of ^18^F-FDG PET metabolic parameters. The sensitivity, specificity, positive predictive value (PPV) and negative predictive value (NPV) were calculated. The data of P < 0.01 for comparison of clinical parameters between nonmetastatic GBC and cholecystitis and ^18^F-FDG PET metabolic parameters with the highest AUC were selected for inclusion in the diagnostic model. Multivariate logistic regression analysis was used to construct a diagnostic model for distinguishing GBC from cholecystitis. The bootstrap test, integrated discriminatory improvement (IDI) and net reclassification improvement (NRI) were calculated for comparison of the diagnostic model and ^18^F-FDG PET metabolic parameters with the highest area under the curve (AUC). The bootstrap test was performed with the *pROC* package, and IDI and NRI were performed with the *PredictABEL* package. Decision curve analyses (DCA) evaluated the clinical utility and accuracy of the ^18^F-FDG PET metabolic parameter and model by calculating the net benefits for a range of threshold probabilities in metabolic parameters with the highest AUC [20]. DCA was performed with the *rmda* package. *P* < 0.05 was considered statistically significant.

## Results

### Baseline characteristics and clinical variables

A total of 122 patients were included in the study, including 88 nonmetastatic GBC patients and 34 cholecystitis patients. Table 1 shows the baseline and clinical characteristics between nonmetastatic GBC and cholecystitis patients. Regarding baseline characteristics, a significant difference was observed in age (65.06 ± 8.34 vs. 59.06 ± 12.25, *P* = 0.019), sex (male:female: 45.5%:54.5% vs. 76.5%:23.5%, *P* = 0.002), smoking history (13 [14.8%] vs. 16 [47.1%], *P* = 0.001) and drinking history (7 [8.0%] vs. 8 (23.5%), *P* = 0.029) between nonmetastatic GBC and cholecystitis patients. According to clinical variables, in the relevant medical history, the proportion of patients with cholecystitis who were diagnosed with gallbladder polyps (3 [3.4%] vs. 5 [14.7%], *P* = 0.038) and cholecystolithiasis (4 [4.5%] vs. 0 [0%], *P* = 0.575) was not significantly different compared with patients with nonmetastatic GBC. In patients' clinical symptoms, there were more patients with fever in cholecystitis than in nonmetastatic GBC (1 [1.1%] vs. 7 [20.6%], *P* = 0.001). There was no significant difference in jaundice (14 [15.9%] vs. 9 [26.5%], *P* = 0.202), abdominal pain (41 [46.6%] vs. 21 [61.8%], *P* = 0.160) or abdominal mass (0 [0.0%] vs. 0 [0.0%], *P* = 0.202) between the two groups. In the laboratory examination, the value of CEA (21.73 ± 69.50 vs. 2.32 ± 1.52, *P* = 0.001) and the proportion of CEA > 5 ng/ml (32 [36.4%] vs. 2 [5.9%], *P* = 0.001) in nonmetastatic GBC were higher than those in cholecystitis. The values of WBC (6.96 ± 3.09 vs. 6.25 ± 2.30, *P* = 0.249), LYM (0.27 ± 0.10 vs. 0.27 ± 0.10, *P* = 0.924), NEUT (0.62 ± 0.12 vs. 0.63 ± 0.11, *P* = 0.714), CA19-9 (937.86 ± 2942.96 vs. 1009.54 ± 3562.21, *P* = 0.910) and the proportion of CA19-9 > 37 U/mL (48 [54.5%] vs. 16 [47.1%], *P* = 0.545) were not significantly different.

**Table 1:** Baseline characteristics and clinical variables between nonmetastatic gallbladder cancer and cholecystitis

	Nonmetastatic gallbladder cancer (*n* = 88)		Cholecystitis (*n* = 34)		*P*-value
**Baseline characteristics**					
Age	65.06 ± 8.34		59.06 ± 12.25		0.019^b^
Sex	40:48		26:8		0.002
(Male:Female, n, %)	45.5%:54.5%		76.5%:23.5%		
BMI	24.46 ± 2.93		23.70 ± 2.93		0.204^a^
Smoking history (n, %)	13 (14.8%)		16 (47.1%)		0.001
Drinking history (n, %)	7 (8.0%)		8 (23.5%)		0.029
**Clinical variables**					
Medical history (n, %)					
Cholecystitis	3 (3.4%)		5 (14.7%)		0.038
Cholecystolithiasis	9 (10.2%)		15 (44.1%)		< 0.001
Gallbladder polyps	4 (4.5%)		0 (0.0%)		0.575
Major signs (n, %)					
Jaundice	14 (15.9%)		9 (26.5%)		0.202
Fever	1 (1.1%)		7 (20.6%)		0.001
Abdominal pain	41 (46.6%)		21 (61.8%)		0.160
Abdominal mass	0 (0.0%)		0 (0.0%)		-
Laboratory examination					
WBC (10^9^/L)	6.96 ± 3.09		6.25 ± 2.30		0.249^b^
LYM	0.27 ± 0.10		0.27 ± 0.10		0.924^b^
NEUT	0.62 ± 0.12		0.63 ± 0.11		0.714^b^
CEA (ng/ml)	21.73 ± 69.50		2.32 ± 1.52		0.001^b^
CEA > 5 ng/ml	32 (36.4%)		2 (5.9%)		0.001
CA19-9 (U/mL)	937.86 ± 2942.96		1009.54 ± 3562.21		0.910^a^
CA19-9 > 37 U/mL	48 (54.5%)		16 (47.1%)		0.545
**Histologic type (n, %)**					
	Adenocarcinoma	78 (89%)	Acute cholecystitis		17 (50%)
	Squamous cell carcinoma	1 (1%)	Chronic cholecystitis		13 (38%)
	Adenosquamous carcinoma	2 (2%)	XGC		4 (12%)
	Others	7 (8%)			

*WBC *white blood cell, *LYM *lymphocyte, *NEUT *neutrophil, *CEA *carcinoembryonic antigen, *CA19-9 *carbohydrate antigen, *XGC *xanthogranulomatous cholecystitis

^a^Student *t* test, ^b^Mann-Whitney test

The pathologic results demonstrated that 78 patients had adenocarcinoma (89%, including 5 patients with malignant transformation of GBC, 1 squamous cell carcinoma (1%), 2 adenosquamous carcinomas (2%) and 7 others (8%, 2 sarcomatoid carcinomas; 3 undifferentiated carcinomas and 2 neuroendocrine carcinomas); 17 had acute cholecystitis (50%); 13 had chronic cholecystitis (38%); and 4 had xanthogranulomatous cholecystitis (12%) (Table 1).

### Comparison of ^18^F-FDG PET metabolic parameters between nonmetastatic GBC and cholecystitis

Compared with patients with cholecystitis, the metabolic parameters of ^18^F-FDG PET in patients with nonmetastatic GBC, including SUVmax (11.62 ± 6.85 vs. 7.62 ± 3.58, *P* = 0.002), SUVmean (6.69 ± 4.15 vs. 4.26 ± 2.04, *P* = 0.002), SUVpeak (9.55 ± 5.90 vs. 5.92 ± 2.66, *P* = 0.001) and SUVR (5.07 ± 3.04 vs. 3.17 ± 1.55, *P* = 0.001), were significantly higher, but there were no differences in TLG (205.76 ± 269.23 vs. 101.67 ± 100.24, *P* = 0.063) or MTV (27.39 ± 29.51 vs. 23.44 ± 19.77, *P* = 0.828). ^18^F-FDG PET metabolic parameters between nonmetastatic GBC and cholecystitis patients are summarized in Table 2.

**Table 2:** Comparison of ^18^F-FDG PET metabolic parameters between nonmetastatic gallbladder cancer and cholecystitis

	Nonmetastatic gallbladder cancer	Cholecystitis	*P*-value
SUVmax	11.62 ± 6.85	7.62 ± 3.58	0.002^b^
SUVmean	6.69 ± 4.15	4.26 ± 2.04	0.002^b^
SUVpeak	9.55 ± 5.90	5.92 ± 2.66	0.001^b^
TLG	205.76 ± 269.23	101.67 ± 100.24	0.063^b^
MTV	27.39 ± 29.51	23.44 ± 19.77	0.828^a^
SUVR	5.07 ± 3.04	3.17 ± 1.55	0.001^b^

*SUVmax* Max standard uptake value, *SUVmean *Mean standard uptake value, *MTV *Metabolic tumor volume, *TLG* Total lesion glycolysis, *SUVR *standard uptake value ratio

^a^Student *t* test; ^b^Mann-Whitney test

### The differential diagnostic performance of ^18^F-FDG PET metabolic parameters and clinical variables in nonmetastatic GBC and cholecystitis

The diagnostic performance is demonstrated in Table 3. The ROC curve showed that SUVR had the highest diagnostic ability among the ^18^F-FDG PET metabolic parameters; the cut-off was 5.9, and the AUC was 0.698 (95% confidence interval [CI]: 0.599–0.796). The results showed that the sensitivity was 0.341 (95% CI: 0.245–0.451), the specificity was 0.971 (95% CI: 0.829–0.998), the PPV was 0.968 (95% CI: 0.815–0.998), and the NPV was 0.363 (95% CI: 0.266–0.471). For clinical variables, its diagnostic ability is relatively low (Table 3). Meanwhile, according to the difference in clinical characteristics between patients with nonmetastatic GBC and cholecystitis, we constructed a diagnostic model based on multivariate logistic regression analysis, and the parameters included fever, cholecystolithiasis, CEA > 5 ng/ml and SUVR. The model showed that the AUC was 0.899 (95% CI: 0.840–0.958), the sensitivity was 0.909 (95% CI: 0.824–0.957), the specificity was 0.735 (95% CI: 0.553–0.865), and the PPV and NPV were 0.899 (95% CI: 0.812–0.950) and 0.758 (95% CI: 0.574–0.883), respectively.

**Table 3:** Differential diagnostic efficiency of ^18^F-FDG PET metabolic parameters and clinical variables and model between nonmetastatic gallbladder cancer and cholecystitis

Parameters	Cut-off	AUC (95%CI)	Sensitivity (95%CI)	Specificity (95%CI)	PPV (95%CI)	NPV (95%CI)
SUVmax	12.5	0.677 (0.577–0.777)	0.398 (0.297–0.508)	0.941 (0.789–0.990)	0.946 (0.805–0.991)	0.376 (0.276–0.489)
SUVmean	7.0	0.684 (0.585–0.783)	0.398 (0.297–0.508)	0.912 (0.752–0.977)	0.921 (0.775–0.979)	0.369 (0.268–0.482)
SUVpeak	9.8	0.690 (0.593–0.787)	0.398 (0.297–0.508)	0.912 (0.752–0.977)	0.921 (0.775–0.979)	0.369 (0.268–0.482)
SUVR	5.9	0.698 (0.599–0.796)	0.341 (0.245–0.451)	0.971 (0.829–0.998)	0.968 (0.815–0.998)	0.363 (0.266–0.471)
Cholecystolithiasis	-	0.556 (0.438–0.675)	0.966 (0.897–0.991)	0.147 (0.055–0.318)	0.746 (0.654–0.820)	0.625 (0.259–0.898)
Fever	-	0.597 (0.477–0.717)	0.989 (0.929–0.999)	0.206 (0.093–0.384)	0.763 (0.672–0.836)	0.875 (0.467–0.993)
CEA > 5ng/ml	-	0.652 (0.553–0.752)	0.364 (0.266–0.474)	0.941 (0.789–0.990)	0.941 (0.789–0.990)	0.364 (0.266–0.474)
Model	-	0.899 (0.840–0.958)	0.909 (0.824–0.957)	0.735 (0.553–0.865)	0.899 (0.812–0.950)	0.758 (0.574–0.883)

Model: Cholecystolithiasis plus Fever plus CEA > 5ng/ml plus SUVR

*CI* Confidence interval, *AUC* Area under the curve, *PPV* Positive predictive value, *NPV* Negative predictive value

The model is shown below.

$$y=\frac1{{}_{1+\mathrm e}-\left(0.49\times SUVR-4.10\times Chelecystolithiasis-3.28\times Fever+4.03\times CEA>5\mathrm{ng}/\mathrm{ml}-0.42\right)}$$

**Fig. 2:**
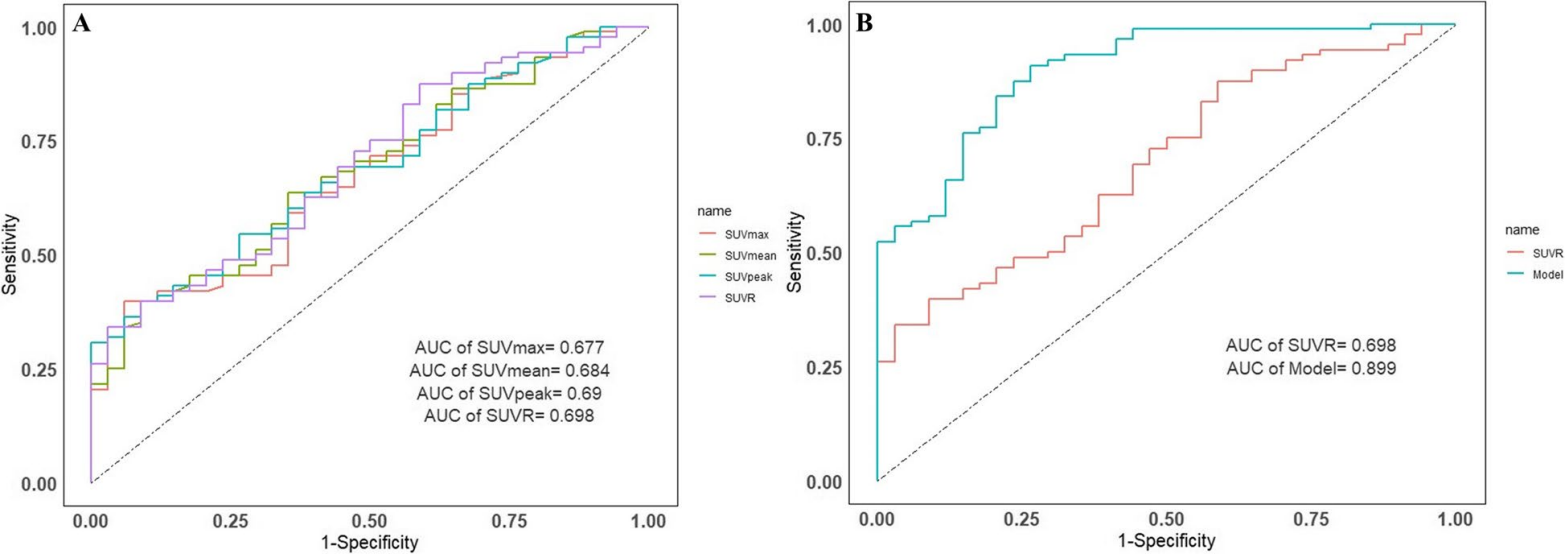
The ROC curves of ^18^F-FDG PET metabolic parameters (**A**) and model (**B**). The areas under the ROC curves for the ability to differentiate nonmetastatic gallbladder cancer from cholecystitis were 0.677 for SUVmax, 0.684 for SUVmean, 0.690 for SUVpeak, 0.698 for SUVR, and 0.899 for the model

The diagnostic efficiencies of the ^18^F-FDG PET parameters and model are shown in Fig. 2 and Table 3.

The addition of cholecystolithiasis, fever and CEA > 5 ng/ml to SUVR allowed a significant reclassification with IDI = 0.369 (95% CI: 0.246–0.491, *P* < 0.001) and categorical NRI = 0.426 (95% CI: 0.160–0.692, *P* < 0.001) compared to SUVR alone. According to the bootstrap test, compared with SUVR alone, the combination of cholecystolithiasis, fever, CEA > 5 ng/ml and SUVR had a statistically significant improvement in ROC (*D* = 0.435; boot: *n* = 2000; boot: stratified = 1; *P* < 0.001) (Table 4).

**Table 4:** Comparison of the SUVR and model to with Bootstrap test, IDI and NRI

Variable	Bootstrap test		IDI	95%CI	*P*	NRI	95%CI	*P*
	***D***	***P***						
Model vs. SUVR	4.35	< 0.001	0.369	0.246–0.491	< 0.001	0.426	0.160–0.692	< 0.001

Model: Cholecystolithiasis plus Fever plus CEA > 5ng/ml plus SUVR

*IDI* Integrated discrimination improvement, *NRI *Net reclassification improvement (categorical), *CI* Confidence interval

### Clinical application

**Fig. 3:**
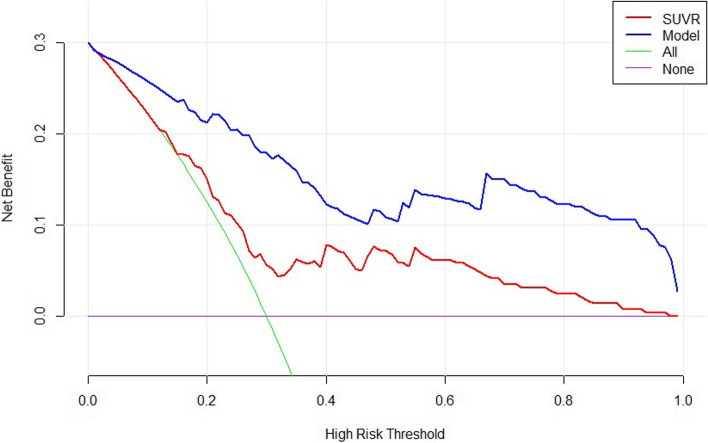
Decision curve analyses (DCA) showed that, regardless of the threshold probability of a doctor or a patient, using the combined model (cholecystolithiasis plus fever plus CEA > 5 ng/ml plus SUVR) in our study to differentiate nonmetastatic gallbladder cancer from cholecystitis was more valuable than using SUVR alone. The x-axis represents the threshold probability, and the y-axis represents the net benefit

The DCA for the SUVR and the model are presented in Fig. 3. DCA showed that the model had a higher overall net benefit than SUVR across the entire range of risk thresholds.

## Discussion

Our research found that ^18^F-FDG PET metabolic parameters have certain value in differentiating nonmetastatic GBC and cholecystitis. Furthermore, diagnostic models based on ^18^F-FDG PET metabolic parameters (SUVR) and clinical variables, including patient signs (fever), medical history (cholecystolithiasis) and laboratory examination (CEA > 5 ng/ml), can effectively help differentiate nonmetastatic GBC and cholecystitis.

Surgical resection is the only possible cure for GBC [21]. Patients with T1a stage tumours can be treated by simple cholecystectomy [22]. After T1a stage, GBC patients who are likely to have tumours removed need radical surgery, including extended cholecystectomy, resection of adjacent liver parenchyma to ensure negative surgical margins, removal of any organs that may be involved in the tumour, and removal of periportal and hepatoduodenal ligaments [21]. Importantly, if only cholecystectomy is carried out for GBC, many patients will have residual disease, and R0 resection is an important prognostic factor [23]. In particular, only a few patients will be suspected of gallbladder cancer before surgery [24], and the others will only be diagnosed after cholecystectomy on histopathological study of the gallbladder specimen [25]. The likelihood of finding residual disease in patients re-explored after incidental discovery of GBC after cholecystectomy has been reported as 38%, 57%, and 77% in patients with T1b, T2, and T3 tumours, respectively [26]. For patients with cholecystitis, the treatment mainly depends on the type of cholecystitis. The main treatment methods for cholecystitis include supportive care and surgical treatment, while surgical treatment is mainly applied to laparoscopic cholecystectomy [21]. Expectant management is the treatment of choice for patients with asymptomatic cholelithiasis [5]. Therefore, judging GBC and cholecystitis before surgery can help patients choose the right treatment. However, the GBC patient's performance is nonspecific, and some of the patient's signs and laboratory examination may coincide with those of cholecystitis patients. The conventional imaging findings of cholecystitis may overlap with those of gallbladder carcinoma [11].

**Fig. 4:**
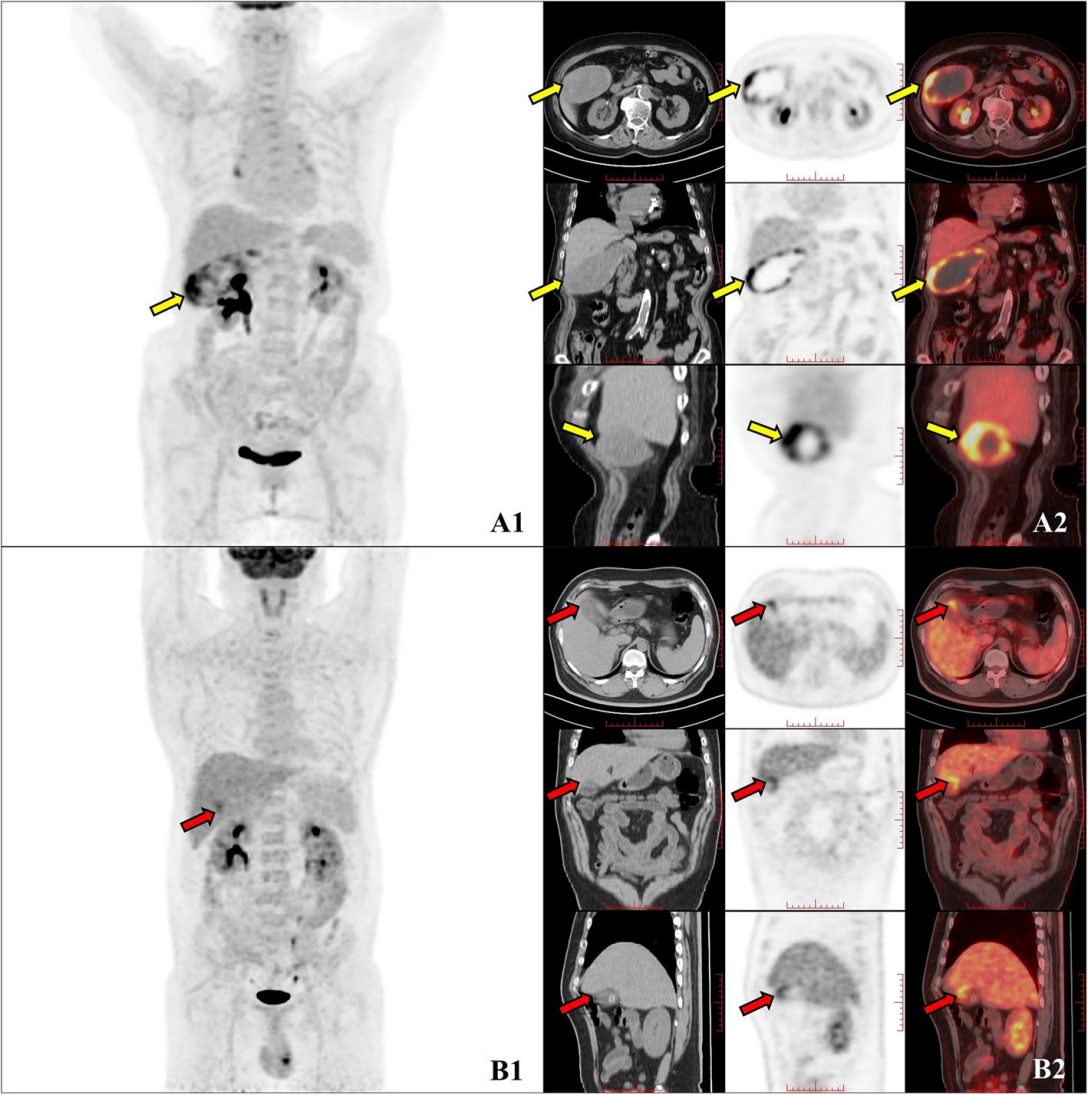
Images A1 and A2 show a 77-year-old woman with medium-differentiated gallbladder adenocarcinoma (yellow arrow). During the physical examination one month prior, the patient was found to have a space-occupying gallbladder, without jaundice, fever, abdominal pain or abdominal mass. The patient had no history of cholecystolithiasis, gallbladder polyps or cholecystitis. The CEA was 6.59, and the CA19-9 was 9.22. The lesion showed that SUVR was 5.5 (A1-MIP image of ^18^F-FDG PET/CT; A2-First line: axial PET, CT, and PET and CT fusion images; Second line: coronal PET, CT, and PET and CT fusion images; Third line: sagittal PET, CT, and PET and CT fusion images). Images B1 and B2 show a 39-year-old man with acute cholecystitis with chronic inflammation of the gallbladder mucosa (red arrow). The patient had no obvious cause of abdominal pain with fever for 1 month. The patient had a medical history of cholecystolithiasis. The CEA was 6.59, and the CA19-9 was 9.22. The lesion showed an SUVR of 2.1 (A1-MIP image of ^18^F-FDG PET/CT; A2-First line: axial PET, CT, and PET and CT fusion images; Second line: coronal PET, CT, and PET and CT fusion images; Third line: sagittal PET, CT, and PET and CT fusion images)

^18^F-FDG PET/CT has been proven to be a very accurate noninvasive tool for evaluating primary tumours in GBC patients [27] and can be used for staging patients with GBC before treatment [12] and evaluating residual lesions after surgery [28]. Therefore, ^18^F-FDG PET/CT is a more accurate noninvasive examination for patients suspected of GBC and metastasis in clinical practice and patients suspected of recurrence after treatment. When ^18^F-FDG PET/CT shows obvious distant metastasis, the diagnosis of gallbladder cancer will be clearer. However, sometimes FDG uptake occurs only in gallbladder lesions. Because benign gallbladder lesions, such as cholecystitis [29], adenomyomatosis [30] and gallbladder polyps [31], also have FDG uptake, when this occurs, the diagnosis is often controversial. The uptake of adenomyomatosis and gallbladder polyps is often lower than that of the liver parenchyma; thus, it is easier to differentiate from GBC [31, 32]. The accumulation of FDG in inflammatory tissues may lead to a false-positive diagnosis of malignant tumours [33]; therefore, it is challenging to judge the characteristics of gallbladder space-occupying lesions with positive FDG uptake, especially for independent gallbladder lesions. Previous studies have disputed the ability of ^18^F-FDG PET in the differential diagnosis of benign and malignant gallbladder lesions [34–37]. Many factors can affect the FDG uptake of gallbladder lesions, such as small lesions, which may lead to false-negatives [34]. Therefore, the patients included in our study were all patients with positive FDG uptake and no distant metastasis found on ^18^F-FDG PET/CT, and all of them received surgical pathology of gallbladder lesions. Our research results showed that, compared with patients with cholecystitis, patients with nonmetastatic GBC have higher FDG uptake (Fig. 4), and SUVR has the best differential diagnostic ability among all metabolic parameters of ^18^F-FDG PET. However, there are still problems in the differential diagnosis of cholecystitis and nonmetastatic GBC using only metabolic parameters. The metabolic parameters show good specificity (range: 0.912–0.971) and are not sensitive enough (range: 0.341–0.398), which may lead to false-negatives.

Cholecystolithiasis is the most important inducing factor of cholecystitis. Blocking the bile duct may induce acute cholecystitis, while recurrent cholecystitis may lead to chronic cholecystitis [9, 38]. Gallstones are an important risk factor for GBC [39], but only one in every 200 gallstone patients has GBC (incidence rate is 0.5%) [4]. Cholecystitis is an inflammatory disease, and there is a possibility of fever in patients. GBC often presents with vague abdominal complaints and systemic signs of anorexia and weight loss [38]. Meanwhile, although the application of serum CEA in the diagnosis of gallbladder cancer is relatively limited, compared with non-GBC, the CEA in gallbladder cancer patients will be higher, with a higher proportion higher than 5 ng/ml [40]. In our study, due to the differences in the signs, medical history and laboratory examination of patients with GBC and cholecystitis, as well as the diagnostic ability of metabolic parameters of ^18^F-FDG PET, we combined clinical variables, including cholecystolithiasis, fever, CEA > 5 ng/ml, and SUVR, to form a differential diagnostic model. Our research results showed that the model has good sensitivity (0.909) and specificity (0.735) in differential diagnosis and has better diagnostic ability than the single application of ^18^F-FDG PET metabolic parameters (SUVR). DCA suggested that the differential diagnosis of nonmetastatic GBC and cholecystitis using the model in our study was more valuable than using SUVR alone, regardless of the physician's or patient's threshold probability. To ensure the reliability of the results, all patients were confirmed by pathology. When a patient is diagnosed with cholecystitis, surgery is not the first choice [38], but if there is an incorrect diagnosis, it is likely to delay treatment. Therefore, our diagnosis model may avoid the above question. This model can help nuclear medicine doctors have a more accurate diagnosis when they encounter independent gallbladder lesions with high FDG uptake and, to a certain extent, help patients choose treatment.

This study had some limitations. First, to our knowledge, this is the first study to apply ^18^F-FDG PET metabolic parameters to the differential diagnosis of nonmetastatic GBC and cholecystitis, and more cases were included compared with other articles that used ^18^F-FDG PET metabolic parameters to differentiate benign and malignant gallbladder. This was a retrospective cohort study, and compared with patients with nonmetastatic GBC, the number of patients with cholecystitis was significantly lower. This may be because some patients did not undergo surgery after clinical diagnosis of cholecystitis and could not obtain pathological results. These factors may lead to statistical bias of the diagnostic model. Second, although no patients with nonmetastatic GBC received tumour-related treatment, some patients with cholecystitis and nonmetastatic GBC received antibiotics after cholecystitis-related symptoms. This may have led to an inaccurate comparison of inflammatory indicators (such as WBC, LYM, and NEUT) in the laboratory examination. However, antibiotic treatment is necessary in some patients with cholecystitis, such as acute cholecystitis [41]. We did not include inflammatory indicators in the diagnostic model, which helps avoid inaccuracies. Third, SUV is influenced by many factors [42], which may lead to nonreproducibility of the model constructed with metabolic parameters. Because ^18^F-FDG PET/CT of patients comes from different machines, the measurement of metabolic parameters may be different due to machine parameters and ^18^F-FDG injection dose. Therefore, we corrected this issue through SUVR to minimize the result bias. Fourth, there were no FDG-negative patients among our cohort. Because these patients were not clinically diagnosed with nonmetastatic GBC, no surgery was performed to obtain pathology. Fifth, some important parameters (such as T stage) were not included in our study due to insufficient medical records. In the future, we will conduct prospective research with a larger sample size and incorporate more variables to improve the diagnostic ability, stability and repeatability of the model.

## Conclusion

In general, ^18^F-FDG PET metabolic parameters are still defective in differentiating nonmetastatic GBC from cholecystitis, but the multiparameter diagnostic model composed of ^18^F-FDG PET metabolic parameters (SUVR) and clinical variables, including patient signs (fever), medical history (cholecystolithiasis) and laboratory examination (CEA > 5 ng/ml), has good diagnostic efficacy in the differential diagnosis of nonmetastatic GBC and cholecystitis. Our results may help judge the characteristics of lesions when solitary high uptake gallbladder lesions are found on ^18^F-FDG PET and provide a more accurate and reliable evaluation for the differential diagnosis of preoperative gallbladder disease, which can avoid unnecessary treatment and surgery.

## Data Availability

The datasets used and/or analyzed during the current study are available from the corresponding author on reasonable request.

## Abbreviations

^18^FDG PET: ^18^F-fluorodeoxyglucose positron emission tomography
AC: Attenuation correction
CEA: Carcinoembryonic antigen
DCA: Decision curve analysis
GBC: Gallbladder cancer
IDI: Integrated discriminatory improvement
LYM: Lymphocyte
MTV: Metabolic tumor volume
NEUT: Neutrophil
NPV: Negative predictive value
NRI: Net reclassification improvement
OSEM: Ordered subset expectation maximization algorithm
PPV: Positive predictive value
SUVmax: Maximum standard uptake value
SUVmean: Mean standard uptake value
SUVpeak: Peak standard uptake value
SUVR: Tumor-to-normal liver standard uptake value ratio
TLG: Total lesion glycolysis
VOI: Volume of interest
WBC: White blood cell
